# Geochemical Modeling of Trivalent Chromium Migration in Saline-Sodic Soil during Lasagna Process: Impact on Soil Physicochemical Properties

**DOI:** 10.1155/2014/272794

**Published:** 2014-07-24

**Authors:** Salihu Lukman, Alaadin Bukhari, Muhammad H. Al-Malack, Nuhu D. Mu'azu, Mohammed H. Essa

**Affiliations:** ^1^Department of Civil Engineering, ACHB, King Fahd University of Petroleum and Minerals, Hafar Al-Batin 31991, Saudi Arabia; ^2^Department of Environmental Engineering, University of Dammam, Dammam, Saudi Arabia; ^3^Environmental Engineering Department, University of Dammam, Dammam 31451, Saudi Arabia

## Abstract

Trivalent Cr is one of the heavy metals that are difficult to be removed from soil using electrokinetic study because of its geochemical properties. High buffering capacity soil is expected to reduce the mobility of the trivalent Cr and subsequently reduce the remedial efficiency thereby complicating the remediation process. In this study, geochemical modeling and migration of trivalent Cr in saline-sodic soil (high buffering capacity and alkaline) during integrated electrokinetics-adsorption remediation, called the Lasagna process, were investigated. The remedial efficiency of trivalent Cr in addition to the impacts of the Lasagna process on the physicochemical properties of the soil was studied. Box-Behnken design was used to study the interaction effects of voltage gradient, initial contaminant concentration, and polarity reversal rate on the soil pH, electroosmotic volume, soil electrical conductivity, current, and remedial efficiency of trivalent Cr in saline-sodic soil that was artificially spiked with Cr, Cu, Cd, Pb, Hg, phenol, and kerosene. Overall desirability of 0.715 was attained at the following optimal conditions: voltage gradient 0.36 V/cm; polarity reversal rate 17.63 hr; soil pH 10.0. Under these conditions, the expected trivalent Cr remedial efficiency is 64.75 %.

## 1. Introduction

In early 1992, a discussion took place between the then Monsanto Chief Executive Officer (CEO) and Administrator of the United States Environmental Protection Agency (USEPA) which ultimately led to the invention of the Lasagna process [1]. In the late 1993, Brodsky and Ho of Monsanto filed the first Lasagna U.S. patent followed by a second one, all published in 1995 [2, 3]. In the Lasagna process, contaminated soil is remediated by creating at least one liquid permeable zone within a contaminated soil region and turning it into treatment zone. Appropriate materials (sorbents, catalytic agents, microbes, oxidants, and buffers) are then introduced into the treatment zone. An electrode is placed at the first end of the contaminated soil region and another of opposite charge is placed at the opposite end of the contaminated soil region. A direct electric current is then transmitted through the contaminated soil region between the two electrodes. This causes movement of water and dissolved organic and inorganic materials in subsurface soils from one electrode (anode) to the other (cathode) under electroosmosis as a result of current movement from anode to cathode. In 1802 electroosmosis was first observed; detailed study of the mechanism was done by Reuss [4] in his classic experiment reported by Abramson [5]. In 1909, Freundlich and Neumann [6] provided the general name “electrokinetic phenomena” to refer to the electrically driven mass flow of dissolved contaminants and pore fluid transport in soils induced by an applied DC voltage. It is made up of transport of pore fluid via electroosmosis (EO) and transport of ions or charged species via electromigration [7]. The direction and quantity of contaminant movement are influenced by the contaminant concentration, solubility, speciation, degree of hydrophobicity, soil type and structure, and the mobility of contaminant ions, as well as the interfacial chemistry and the conductivity of the soil pore fluid [8]. The remedial efficiency generally depends on the nature of the contaminants and soil properties, such as pH, permeability, adsorption capacity, buffering capacity, and geochemical processes (such as acid/base reactions and migration, dissolution/precipitation, redox reactions, complexation, and speciation) [7, 9]. Saline-sodic soils possess electrical conductivity above 4 dS/m, soil paste pH greater than 8.2, and exchangeable sodium percentage greater than 15 [10, 11].

First application of electrokinetics took place in India in the 1930s. It was used to remove excess salts from alkali soils in order to restore it to arable condition [13]. Following its invention in 1993, extensive studies started in 1994 in bench-scale [14] then scaled up in a pilot-scale under laboratory conditions. The first field test called “Phase I: Small Field Test” was conducted in 1995 at the Paducah gaseous diffusion plant (PGDP) site whose soil was contaminated with trichloroethylene (TCE). Full-scale remediation using the Lasagna technology was undertaken at two other contaminated sites in the United States [15]. The specific details of all the Lasagna process implementations are presented in Tables 1 and 2. It is noteworthy that only two of the studies have considered simultaneous removal of contaminant mixture. All others have dealt with only a single organic compound or heavy metal. It has already been observed that contaminated soils do not contain single contaminants only but usually several pollutants appear in the soil in mixed components [16–19].

**Figure 1:**
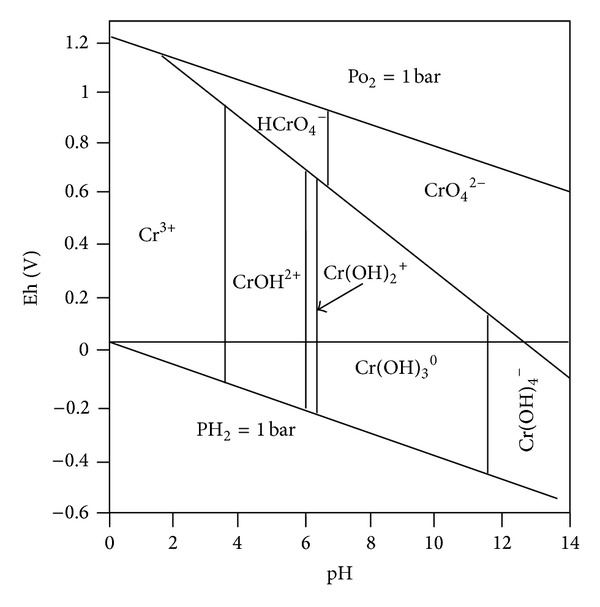
Redox potential (Eh)-pH diagram for Cr–O–H system [12].

The geochemical properties of the most stable forms of Cr, that is, trivalent and hexavalent Cr under electrokinetic remediation, have been extensively studied in different types of soils (kaolin, glacial till, etc.) by Reddy and his coworkers and other investigators [28–36]. The trivalent Cr, though considered relatively nontoxic compared to the hexavalent Cr, exists in the subsurface environments as cation, Cr^3+^, and in the following hydroxocomplex forms: Cr(OH)_4_^−^, CrOH^2+^, and Cr(OH)_3_^0^. Cr^3+^ and CrOH^2+^ ions are mostly prevalent at soil pH values less than 6, while Cr(OH)_4_^−^ and Cr(OH)_3_^0^ ions prevail when pH is greater than 11.8. The redox state also affects the Cr state with reduced state favoring the presence of the trivalent Cr while the oxidized state favors the existence of the hexavalent Cr. Most of the trivalent Cr species are less mobile because of their low solubility over wide pH range (<12) and may be readily adsorbed by the negatively charged clay surfaces. There exists redox state in the subsurface environment because of the generation of oxygen and hydrogen gases at the electrodes in addition to the possible presence of iron (reducing agent), manganese (oxidizing agent), or microorganisms. The redox potential (Eh) and soil pH determine the possible oxidation of Cr from the trivalent to hexavalent form as shown in Figure 1. Chinthamreddy and Reddy [28] have found no significant oxidation of trivalent Cr in high buffering capacity soil such as glacial till.

Empirical modeling using response surface methodology (RSM) offers great and numerous advantages which include large amount of information from a small number of experiments, evaluation of simultaneous interaction effects of the independent parameters on the responses, and simultaneous optimization of multiple factors and responses for obtaining optimal conditions [37, 38]. The key success of RSM is uncovering interactions of factors which cannot be achieved using the traditional one-factor-at-a-time (OFAT) optimization approach [39]. Fundamental understanding of the physics and chemistry which governs the process is essential in determining the influential factors to be investigated and their levels or ranges are necessary for successful implementation of RSM for any process modeling and optimization. Basically, there exist four different experimental designs for RSM implementation: 3-level factorial design (3FD), Box-Behnken design (BBD), central composite design (CCD), and Doehlert design (DD). Bezerra et al. [38] have reviewed each of these design methods. The Box-Behnken design is obtained by combining two-level factorial designs with incomplete block designs followed by adding a specified number of replicated center points. BBD is preferred when investigating three (3) factors using RSM, because it will give enough information for analyzing factor-response interactions from the least experimental runs when compared to 3FD and CCD.

Some microbially-driven biotransformation processes may affect the soil physicochemical properties after electrokinetic remediation because of the passage of electric current and development of pH gradients [40]. These lead to original soil mineral degradation and alteration via biotransformation. While biotransformation deals with the bioweathering and alteration or degradation of clay minerals, biomineralization refers to the formation of amorphous and crystalline materials from aqueous ions by biologically mediated processes. In addition to current and pH gradients, heavy metals also cause to affect the following biological assays: soil microbial biomass carbon, enzyme activity, basal soil respiration, and earthworm assays and seed assays [41–44]. Given the aforementioned intricacies of the geochemical behavior and migration of trivalent Cr in soil during electrokinetic remediation, this study was aimed at investigating trivalent Cr migration and remedial efficiency in high buffering capacity and alkaline soil during electrokinetic study in addition to the impacts of the soil remediation on the physicochemical properties of the soil. A carefully designed experiment using BBD was used to study the interaction effects of voltage gradient, initial contaminant concentration, and polarity reversal rate on the trivalent Cr remedial efficiency in saline-sodic soil that was artificially spiked with Cr, Cu, Cd, Pb, Hg, phenol, and kerosene using RSM modeling and optimization tools.

## 2. Materials and Methods

### 2.1. Characterization

Natural saline-sodic clay, obtained from Al-Hassa Oasis, Saudi Arabia, was used in this study. The soil has the following characteristics: pH (8.3), moisture content (3.91%), soil organic matter (2.59%), electrical conductivity (15.24 dS/m), specific surface area (9.07 m^2^/g), pore volume (0.014 cm^3^/g), pore size (62.55 Å)—mineralogy from X-ray diffraction (XRD), quartz (SiO_2_) (87.4%), calcite (CaCO_3_) (5.2%), and dolomite (CaMg(CO_3_)_2_) (7.4%). X-ray fluorescence spectroscopy (XRF) revealed that the soil consists of the following elements: Ca (37.64%), Si (34.73%), Fe (10.41%), Al (7.6%), K (3.42%), Mg (2.48%), Pd (2.85%), and Ti (0.86%). These properties were determined using methods of the American Society of Testing and Materials (ASTM) standards and were reported elsewhere [45]. The granular activated carbon (GAC) used in the present study whose surface area is 952 m^2^/g was produced locally from date palm pits using phosphoric acid impregnation method. Its characterization and properties have been reported elsewhere [46, 47].

### 2.2. Adsorption Testing

Single and competitive adsorption of five heavy metals (Cr, Cd, Cu, Zn, and Pb) were performed to determine the selectivity sequence and to understand the adsorption behavior of these metals under different pH conditions. This is particularly important to this study, because soil mineralogy affects heavy metal adsorption behavior and selectivity sequence under different pH conditions. Lukman et al. [45] reported the detailed procedures carried out for the competitive adsorption testing.

### 2.3. Coupled Electrokinetics-Adsorption Study

Fifteen (15) bench-scale experiments, each having a 21-day run time, were designed and performed to investigate the migration and distribution of trivalent Cr in a contaminant mixture using the coupled electrokinetics-adsorption technique and to understand the operating variables' effects on saline-sodic soil.

### 2.4. Reactor Design and Experimental Procedures

**Figure 2:**
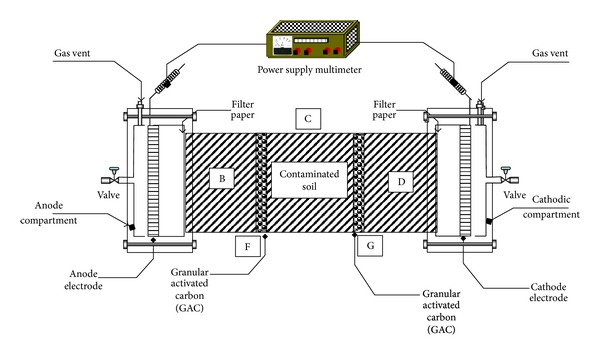
Coupled electrokinetics-adsorption experimental setup.

The Plexiglas reactor total volume was about 2268 cm^3^, made of seven chambers. The overall reactor dimensions are 24 cm (long) × 10 cm (width) × 12 cm (depth). Approximately 1 kg of local KSA soil was artificially spiked with kerosene, heavy metals (Cu, Cr, Cd, Pb, Zn, and Hg), and phenol at predetermined concentrations. Thorough mixing was done using mechanical mixer (Gilson Company Inc.) so as to achieve a homogeneous distribution of the contaminants around the soil matrix. The mixed spiked soil was placed in a fume-hood for drying over a period of time necessary to evaporate the solvents (hexane and distilled water). Distilled water was added to adjust the final moisture content of the soil to about 33–70%. The initial conditions of the soil pH, moisture content, organic matter, and electrical conductivity were measured as well as the actual initial concentrations of the contaminants. Then, the uniformly mixed contaminated soil was placed into the cell layer by layer. Each layer was compacted with stainless steel spatula so that the amount of void space was minimized. The reactor used for the experiments consists of the cell, two graphite electrodes serving as anode and cathode, DC power supply (LG, GP-505), processing fluid reservoirs, heavy duty recirculation pump (BVP Instratec), portable data logger (TDS-303, Tokyo Sokki Kenkyujo Co., Ltd) for real-time monitoring of temperature, electric current, and voltage across the system (Figure 2). The two electrode compartments with 240 mL working volume, placed at each end of the cell, were isolated from the soil zone by a porous Perspex plate and filter paper. The conditioning of the electrolyte was controlled using anolyte (2N NaOH) and catholyte (1N HNO_3_). The pH of the processing fluids was monitored every 8 hr for the 21-day duration of each test. Based on the pH and volume the processing fluids remaining in the electrode chambers, complete replacement, or refill were carried out accordingly. Two planar-shaped electrodes, 10 cm × 10 cm × 0.5 cm, were used to generate a uniform electric field. Within the described cell, two treatment zones that cut across the cell vertically bracketing the spiked soil compartment were filled with the GAC. The data monitoring system was recording electric current variation, applied voltage, and temperature of the soil compartments online following a 30 min preset time step and automatically stores them for subsequent retrieval using floppy disc which can be read using personal computer for easy data and energy consumption analysis. The power supply provides a constant DC electric voltage for the electrokinetic tests. Every week, fractions of the soil specimens were taken at the center of each chamber to determine the residual concentrations of the contaminants, soil pH, water content, organic matter, and electrical conductivity. Upon the completion of each test, the electrode assemblies were disconnected and the soil specimen was extruded from the cell, sectioned into parts, weighed, and preserved in glass vials for organic extraction, heavy metal digestion, and subsequent analyses using the analytical procedures outlined below.

### 2.5. Analytical Procedures for Contaminant Extraction and Analysis

*Heavy Metals*. Extraction of heavy metals from soil samples was performed according to guidelines spelt out in EPA method 3050B for acid digestion of soils, sediments, and sludges [48] and analyzed using flame atomic absorption spectrometry (AAnalyst 700, Perkin Elmer). All soil samples were extracted in duplicate. EPA method 7000B [49] was employed for heavy metal analysis using flame atomic absorption spectrometry except for mercury which was analyzed using mercury analyzer (Solid Mercury Analyzer SMS 100, Perkin Elmer) according to EPA method 7473 [50]. Visual MINTEQ 3.0 [51] was employed to model the metal ion speciation using its dissolved concentration, pH, temperature, and ionic strength.

*Kerosene and Phenol*. A mixture of methylene chloride and hexane (1 : 1) (v/v) was used as the extraction solvent. Soil samples were extracted using pressurized fluid extraction according to EPA method 3545 procedures [52] using accelerated solvent extractor (ASE 200, Dionex). Volume of extract generated was then injected into the GC-MS (Clarus 580, Perkin Elmer) equipped with autosampler for analysis. TPH quantification was done by using the total chromatographic area counts using retention time range for the elution of hydrocarbon within the kerosene range C_8_–C_16_. Guidelines spelt out in EPA mMethod 8270D [53] for the quantification of semivolatile organics by GC-MS were adhered to.

#### 2.5.1. Data Reliability: QC Protocols, Accuracy, and Precision

To evaluate reliability of the analytical procedures, duplicate samples were analyzed for each sample. Quality control (QC) protocols spelt out in EPA method 7000B [49] were used. These include the use of initial calibration blank (ICB), initial calibration verification (ICV), continuous calibration verification (CCV), and continuous calibration blank (CCB). The accuracy of the spiked soil samples was evaluated using percent recovery set at about ±30% of the spiked value [49]. Repeatability of the experimental results was assessed by ensuring that the precision obtained using the relative percent difference (RPD) was not above 30%.

### 2.6. Testing Program and Mathematical Model Development

Box-Behnken design (BBD) was chosen for the experimental design because of its advantages over central composite design (CCD) and 3-level factorial design when dealing with only three factors. In BBD, the experimental points are hyperspherically arranged, equidistant from the central point [38]. Response surface methodology (RSM) was used in modeling, optimization, and interpretation of the results with the help of Design-Expert version 8 (Stat-Ease, Inc.) platform [39, 54]. The investigated variables (called factors in RSM) are the polarity reversal rate, voltage gradient, and initial contaminant concentration designated as A, B, and C, respectively. These variables were selected based on their known influence on contaminant remedial efficiency and were coded and varied according to Table 3. Based on the factor levels and the chosen number of central points (3), a total of fifteen (15) experiments were randomly designed, using the BBD (Table 4), and subsequently conducted. Only one central point is shown in Table 4.

This experimental design was preliminarily evaluated using variance inflation factor (VIF) to check for orthogonality (independence of factors) and leverage which quantitatively measures the potential of a design point to have significant influence on model fit [54]. These were determined using (1) and (2), respectively. VIF value of 1 indicates that the factor is orthogonal to all other factors in the design. In a case whereby factors are highly correlated, then,* R* ^2^ value becomes a poor indicator of model's predictive ability and it becomes more and more difficult to unravel how each of the investigated factors affect the response. Experimental points having high leverage values close to 1 should influence the model fit by carrying any error (experimental or measurement) into the model; as such, they should be conducted more carefully [39]:

(1) $$\begin{array}{c} \text{VIF}=\frac{1}{\left(1-R_{i}^{2}\right)} \end{array}$$

(2) $$\begin{array}{c} \text{Leverage}=\frac{p}{n}, \end{array}$$

where *R*_*i*_^2^ is the coefficient of determination; *p* is the number of model terms; and *n* is the number of experiments.

Following design evaluation, the responses were fitted to a quadratic model which was fine-tuned by removing any insignificant term. This will maximize* R* ^2^ and minimize lack of fit. The general quadratic equation for fitting models in RSM is

(3) $$\begin{array}{c} y=\;\beta_{o}+\;\overset{k}{\underset{i=1}{\sum }}\beta_{i}x_{i}+\;\overset{k}{\underset{i=1}{\sum }}\beta_{ii}x_{i}^{2}+\;\overset{k}{\underset{1\leq i\leq j}{\sum }}\beta_{ij}x_{i}x_{j}+\varepsilon , \end{array}$$

where *y* is the response or dependent variable; *k* is the number of factors; *β*_*o*_, *β*_*i*_, *β*_*ii*_, and *β*_*ij*_ are the coefficients to be fitted using regression for constant term, linear, quadratic, and interaction parameters, respectively; and *x* is the variables.

The developed models were evaluated using the rich diagnostic tools provided in Design-Expert which include normal plot of residuals (to test the assumption of normality of residuals), predicted versus actual plot (to test the assumption of constant variance), Box-Cox plot (to check the need for data transformation), and externally studentized residuals (to check the presence of any outlier in the data). The effects of factors were compared at a particular point in the design space using the perturbation plot. Response surface and contour plots were then generated.

### 2.7. Use of Desirability Function in Numerical Optimization

Desirability function, being one of the mathematical methods for computation of critical values (maximum or minimum) and measuring overall success of optimizing multiple responses using geometric mean, was employed for the optimization of trivalent Cr remedial efficiency. A search for a combination of factor levels which simultaneously satisfies the goals imposed on factors and responses is first carried out, followed by combining these goals into an overall desirability function that ranges from 0 (outside of the optimization limits) to 1 (at the goal). Combining all responses into overall desirability eliminates favoring one response over another. The aim is not to clinch a desirability value of 1 but to find a good set of conditions that will meet all the set goals for each factor and response [55]. This is achieved using numerical optimization algorithms [52].

## 3. Results and Discussion

Discussion of the monitored results obtained after performing thirteen (13) tests with 3 centre points will be focused on geochemical processes affecting sorption/desorption and migration/removal mechanisms such as the development of acid/base fronts, migration and reactions, dissolution/precipitation, oxidation/reduction reactions, complexation, and metallic ion speciation. In addition, presentation of the developed mathematical models and discussion on how the factors affect the respective responses will follow.

### 3.1. Single and Competitive Adsorption of Heavy Metals on Clay

Lukman et al. [24, 45] have discussed the physicochemical characteristics of the saline-sodic soil. Additional discussion will be provided in the subsequent sections. Lukman et al. [45] have found out that the adsorptive capacities of Cu and Zn ions are higher in the multicomponent adsorption scenario than in the single component scenario. The adsorption selectivity sequences obtained using the coefficient of distribution for the single and multicomponent scenarios are Cr > Pb > Cu > Cd > Zn and Cr > Cu > Pb > Cd > Zn, respectively [45]. Yong et al. [40] have identified the general factors that influence selectivity sequence to be ionic size or activity, first hydrolysis constant, soil type, and pH of the system. From the multicomponent desorption study, trivalent Cr ions were tightly held by the soil surface, thus having the least percentage desorption, followed by Cd and Cu ions. Reddy and his coworkers [28, 30, 32] have reported that trivalent Cr ions adsorb highly to soil solids and form cationic species that are insoluble over a wide range of pH. This is in line with the present findings by Lukman et al. [45] which revealed high selectivity for the trivalent Cr during multicomponent adsorption and desorption tests.

### 3.2. Soil pH Distribution, Electrical Conductivity, Bipolar Effects, Electroosmotic Flow, and Current

*Soil pH and Electrical Conductivity*. The soil pH (8.3) indicates that it contains appreciable soluble salts capable of undergoing alkaline hydrolysis such as sodium carbonate [11]. The hydrolysis of calcite and dolomite may be limited by their low solubility, thus producing a pH of about 8–8.2 in soils. In addition, Na^+^ ions do not strongly compete with H^+^ ions for exchange sites as do Ca^2+^ ions that are strongly and more tightly held on the soil surface. The inability of the displaced Na^+^ ions to inactivate OH^−^ ions results in increased soil pH, which is usually greater than 8.2. Moreover, for a soil whose pH is greater than 8.2, its exchangeable sodium percentage has to be greater than 15 [11]. Presence of calcite and dolomite coupled with alkaline hydrolysis of sodium carbonate gives high electrical conductivity to the soil (15.24 dS/m).

The saline-sodic nature of the soil necessitates the use of processing fluids (2N NaOH and 1N HNO_3_) to continuously neutralize the rapidly generated H^+^ and OH^−^ ions at the anode and cathode, respectively. These fluids were monitored every 8 hours and replaced as they degraded. HNO_3_ and NaOH are strong acid and base, respectively, and dissociate completely according to the following reactions:

(4) $$\begin{array}{c} \text{HN}{\text{O}}_{3}\left(\text{l}\right)⟶{\text{H}}^{+}\left(\text{aq}\right)+{\text{N}{\text{O}}_{3}}^{-}\left(\text{aq}\right) \end{array}$$

(5) $$\begin{array}{c} \text{NaOH}\left(\text{aq}\right)⟶\text{N}{\text{a}}^{+}\left(\text{aq}\right)+\text{O}{\text{H}}^{-}\left(\text{aq}\right). \end{array}$$

Because of the electrochemical decomposition of water, OH^−^ and H^+^ ions are produced at the cathode and anode, respectively, as shown in (6) and (7):

(6) $$\begin{array}{c} 4{\text{H}}_{2}\text{O}\left(\text{l}\right)+4{\text{e}}^{-}⟶{\text{H}}_{2}\left(\text{g}\right)+4\text{O}{\text{H}}^{-}\left(\text{aq}\right) \end{array}$$

(7) $$\begin{array}{c} 2{\text{H}}_{2}\text{O}\left(\text{l}\right)⟶{\text{O}}_{2}\left(\text{g}\right)+4{\text{H}}^{+}\left(\text{aq}\right)+4{\text{e}}^{-}. \end{array}$$

The electrochemically generated H^+^ and OH^−^ ions due to water electrolysis at the anode and cathode, respectively, are neutralized to form water molecules (8) because of the OH^−^ and H^+^ ions produced from the dissociation of the catholyte and anolyte, respectively, as shown in (5) and (4):

(8) $$\begin{array}{c} {\text{H}}^{+}\left(\text{aq}\right)+\text{O}{\text{H}}^{-}\left(\text{aq}\right)⟶{\text{H}}_{2}\text{O}\left(\text{l}\right). \end{array}$$

**Figure 3:**
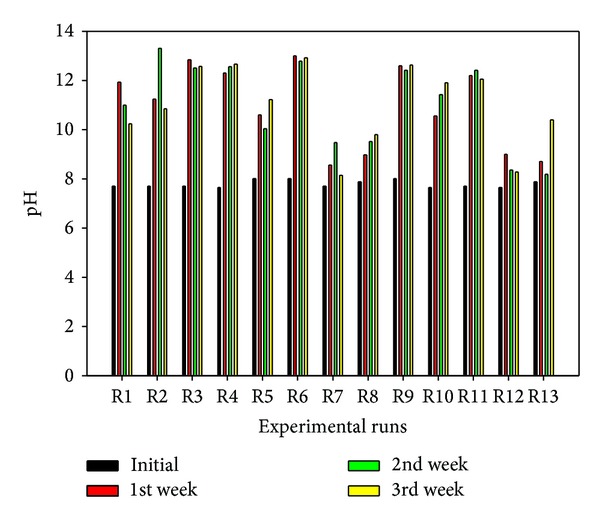
Weekly pH variation.

**Figure 4:**
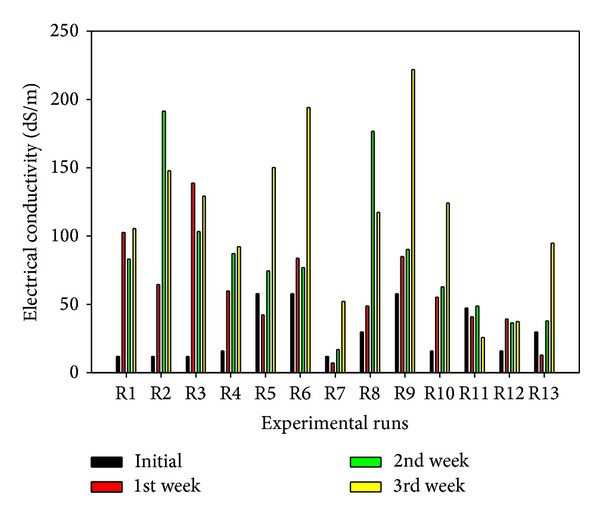
Weekly soil electrical conductivity variation.

**Figure 5:**
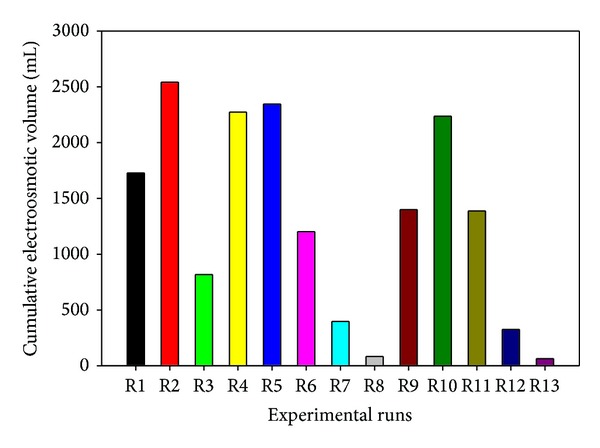
Cumulative electroosmotic volume for each test.

The oxygen and hydrogen gases generated may be vented out, while some amount may go into the soil and alter the redox chemistry [56]. Na^+^ and NO_3_^−^ ions migrate into the soil to the opposite electrodes thereby increasing the electrical conductivity as the treatment process progresses. A sustained and variable electroosmotic flow was observed due to the migration of the Na^+^ ions, which could enhance the migration of the double layer complexes toward the cathode, while nitrate ions could be involved in complex formation with the cations [28]. This electroosmotic flow will lead to decreasing volume of the anolyte and increasing volume of the catholyte over time. Hence, refilling the anolyte is necessary if it has not degraded completely. In addition, since the processing fluids are finite in volume and the electrochemical decomposition of water at the electrodes is continuous for the test duration, then, a time will be reached when all the ions in the processing fluids have been exhausted. Consequently, rise and fall in catholyte pH and anolyte pH, respectively, are expected before the complete replacement of the processing fluids. Now, OH^−^ ions generated at the cathode according to (6) migrate into soil toward the anode. In this migration process, soil pH rises (Figure 3) and metal hydroxides are formed which could precipitate and reduce the electrical conductivity (Figure 4) and increase current consumption near the cathode [41]. At the same time, soluble hydroxocomplexes are formed with the cations due to complexing property of the hydroxyl ions [24, 57]. On the other hand, hydrogen ions generated at the anode (7) migrate toward the cathode. This process may lead to soil protonation or desorption of indigenous and spiked heavy metals and hence increase the electrical conductivity (Figure 4) [32]. Given the presence of calcite and dolomite in the soil minerals, the developing acid front may be buffered by the carbonate mineral, thereby hindering any fall in the soil pH (Figure 3). From the forgoing discussion, it is clear that there will be an overall increase in the soil pH and electrical conductivity (Figure 4) as the integrated electrokinetics-adsorption remediation progresses. Results obtained for electrokinetic remediation of high buffering capacity glacial till by Reddy and hiscoworkers [30–32, 58] have corroborated these findings. The transient nature of the acid/base front migration and reactions may be responsible for the lower final values of some pH and EC than the preceding 1st or 2nd week values. In addition, the electroosmotic flow (Figure 5) will undoubtedly vary spatially and temporally as it also depends on the soil zeta potential, processing fluids pH, pore fluid viscosity, and permittivity [7, 59–61].

**Figure 6:**
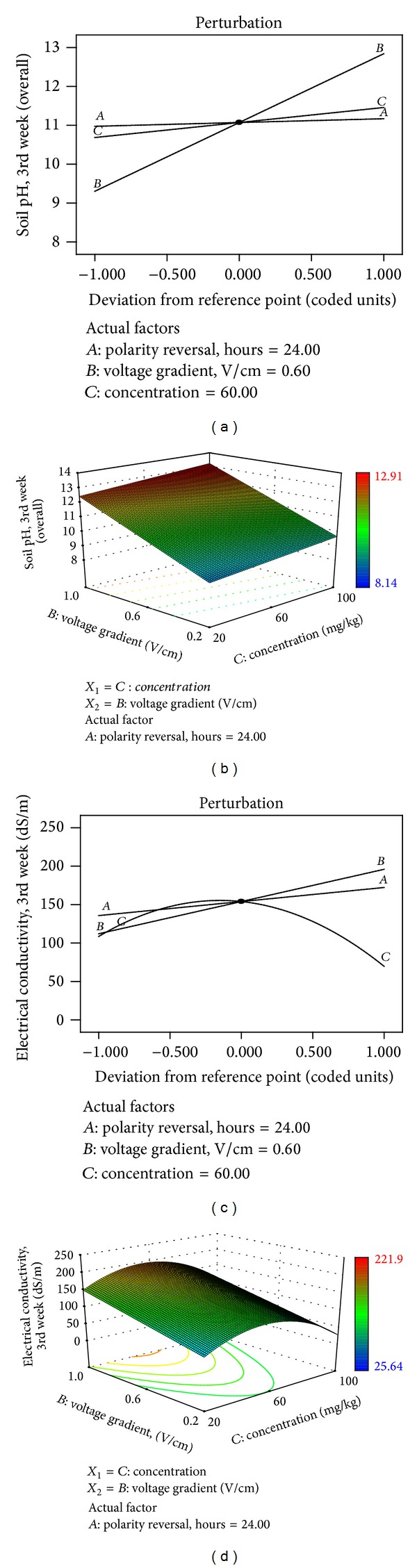
Perturbation plots showing the relative significance of factors on soil pH (a) and electrical conductivity (c) (left). 3D response surface and contour plots showing how the influential factors affect soil pH (b) and electrical conductivity (d) (right).

It was observed from Figure 3 that the average initial soil pH after spiking is within the range 7.7–8, lower than the original soil pH (8.3), while the final pH ranges from 8 to 12.9. The lower initial pH was due to the acidity of the contaminant solutions, while the higher final pH values resulted from the high buffering capacity of the soil which neutralized the generated acidic front from (7) but allowed the migration of the basic front generated from (6). In addition, the hydroxyl ions generated from the dissociation of NaOH (5) and reduction of water at the cathode (6) aid in neutralizing the generated acidic front. Consequently, all weekly pH values are higher than the initially spiked soil pH for all the tests. Additionally, low pH rise (8–10.4) was observed for all the tests conducted using 0.2 V/cm (R7-8, R12-13) whereas highest pH (12.6–12.9) was recorded for all tests conducted using 1 V/cm (R3-4, R6, and R9) consistently. High voltage gradient leads to the passage of high amount of current which increases the rate of the electrochemical decomposition of the electrolyte and enhances subsequent migration of the basic front into the soil. This basic front migration is responsible for raising the soil pH. This observed effect of the voltage gradient on the soil pH has been successfully modeled mathematically and the coded linear model equation at 5% significant level (0.05 *P* value) is presented in (9) while the graphical presentation of the significant influential factors together with 3D response surface and contour plots is given in Figures 6(a) and 6(b):

(9) $$\begin{array}{c} \text{Soil}\;\text{pH}=11.07+0.097*A+1.77*B+0.39*C, \end{array}$$

where* A* is the polarity reversal, hr;* B* is the voltage gradient, V/cm; and* C* is the concentration, mg/kg.

Anderson and Whitcomb [39] have reported that* R* ^2^ is biased; hence, a more accurate, less biased, and better goodness-of-fit statistic called adjusted* R* ^2^ was computed for evaluating the model accuracy. The model's* R* ^2^ and adjusted* R* ^2^ (unbiased estimate of the coefficient of determination) are 0.7725 and 0.7105, respectively. High values of* R* ^2^ are essential for modeling the experimental design space, while in identification of significant factors* R* ^2^ value does not matter and for significant factors will remain significant [39]. It is very clear that model equation, perturbation, and 3D response surface plots have shown the significant influence of voltage gradient on the soil pH over the other factors (polarity reversal rate and initial contaminant concentration). The relative contribution or effect of any given model term is directly proportional to its coefficient. Perturbation plot (Figure 6(a)) revealed a sequence of relative influence of the operating parameters on the target response as follows: voltage gradient > concentration > polarity reversal.

**Figure 7:**
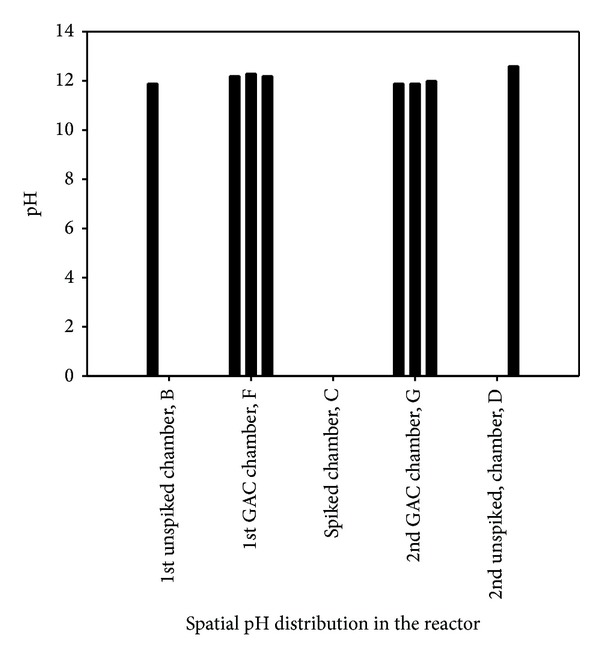
pH profile with two GAC treatment zones for investigating bipolar effects (R11).

*Bipolar Effects*. The two treatment zones F and G contain 100% granular activated carbon which may be used as electrode material due to its electrical conducting properties [14]. The sides of the GAC chambers facing anode and cathode electrodes tend to behave as bipolar electrodes by acting as cathode and anode while the inner sides behave as anode and cathode, respectively. These bipolar electrodes would be expected to generate H^+^ and OH^−^ ions depending on whether the side is acting as anode or cathode [20] and may be expected to alter the pH distribution in the soil profile. These bipolar effects were investigated at the end of R11 and the pH profile is presented in Figure 7. The pH profile shows the variation of pH within the unspiked chambers B and D, spiked chamber C, and GAC chambers F and G. The pH ranges from 11.9 (near the anode) to 12.6 (near the cathode) which suggest that bipolar effects did not manifest due to the presence of carbonate minerals that impact high acid buffering capacity.

Sparks [62] posited that electrical conductivity (EC) is the best index for the assessment of soil salinity. As important as this parameter is, most works on electrokinetic remediation failed to at least report the soil electrical conductivity, let alone monitor its variation over the treatment duration. Electrical conductivity greatly influences electrokinetic remediation, because it determines the amount of current flowing through the soil. The usual voltage gradient of 1 V/cm for bench-scale studies [63] when applied to saline-sodic soils would lead to high electric current flow. Lukman et al. [24] have reported that this would lead to excessive soil heating, reduction in the soil moisture content, high energy and process fluid consumption, high electroosmotic flow rate (Figure 5), and in some cases higher percentage removal of contaminants. EC is simultaneously influenced by many soil properties, viz; water content, soluble salts, grain size, humus, temperature, texture, and cation exchange capacity (CEC) [64]. The 1st week of EC data shows that tests conducted using 1 V/cm (R3, 9, 6) possess the highest EC values with R1 (0.6 V/cm) coming second highest. No discernible trend was visible in the case of initial contaminant concentration despite its influence on the EC as depicted in Figure 6(c). Similar trend was observed for the 3rd week, where R9 and 6 have the highest EC values (Figure 4). A general increase of EC with time and voltage gradient (Figures 6(c) and 6(d)) was observed (except for R11). The reason for this observation has been elaborately discussed above. These variations and impacts of the influential investigated factors have been modeled and presented in the 3D response surface plot in Figure 6(d). Perturbation plot (Figure 6(c)) revealed a sequence of relative influence of the operating parameters on the soil electrical conductivity as follows: concentration > voltage gradient > polarity reversal.

*Electroosmotic Flow*. The cumulative electroosmotic volume for all the tests presented in Figure 5 shows that R2 (20 mg/kg), R5 (60 mg/kg), and R4 (100 mg/kg) have the highest values. Other parameters that may influence electroosmotic flow are clay zeta potential, voltage gradient, and time-dependent fluid properties such as dielectric constant and viscosity [65]. Equation (10) shows the electroosmotic velocity as derived according to Helmholtz-Smoluchowski (H-S) theory:

(10) $$\begin{array}{c} v_{e}=\frac{\varepsilon_{s}\zeta }{\eta }E=k_{e}E, \end{array}$$

where* v*_e_ is the electroosmotic velocity; *ε*_*s*_ is the pore fluid permittivity;*η* is the pore fluid viscosity;*ζ* is the soil zeta potential; *k*_*e*_ is the coefficient of electroosmotic conductivity; and* E* is the voltage gradient.

**Figure 8:**
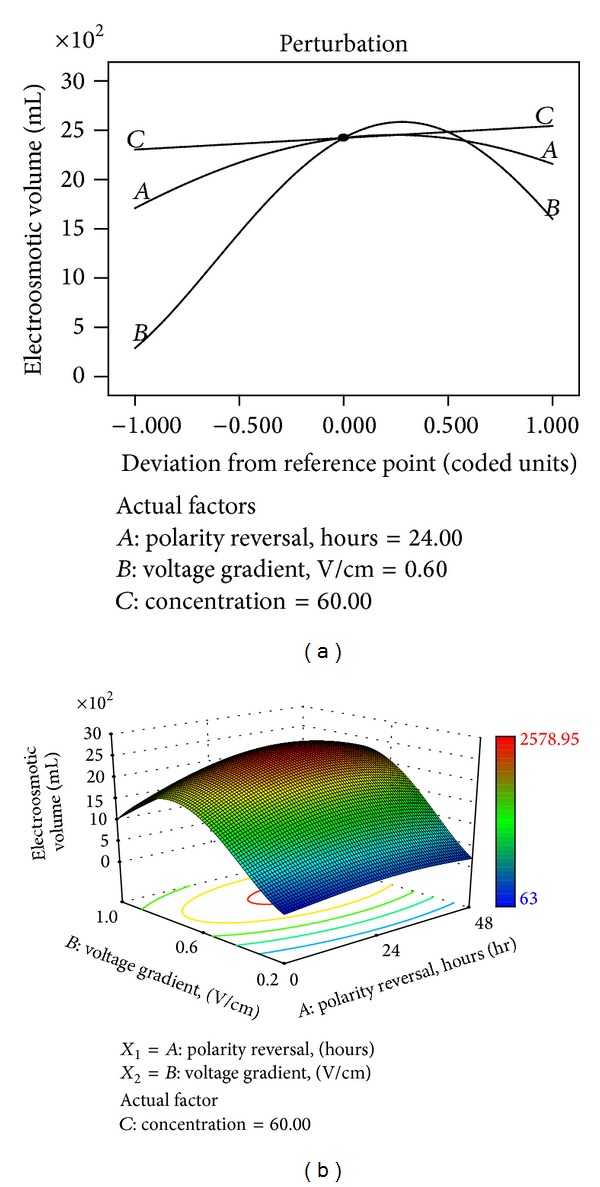
(a) Perturbation plot showing the relative significance of factors on electroosmotic volume. (b) 3D response surface and contour plots showing the influence of voltage gradient on cumulative electroosmotic volume.

These parameters make the measured electroosmotic volume for all the tests to vary temporally. The reduction of the thickness of the diffuse double layer resulting from higher ionic concentration with subsequent higher ionic strength causes reduction in the electroosmotic flow [66]; hence higher concentrations usually yield lower electroosmotic volume (Figure 5). Reddy et al. [66] have observed similar trend. The electroosmotic volume usually decreases with time, because of the increase in electrical conductivity with time (Figure 4) that leads to higher ionic strength as the treatment proceeds. Moreover, voltage gradient has been observed to be most influential to the electroosmotic flow (Figure 8). The least electroosmotic volumes recorded belong to the lowest voltage gradient used (0.2 V/cm), that is, in the case of R7, R12, R8, and R13. This is because high voltage gradient causes the passage of high electric current, which leads to high electromigration with subsequent substantial transfer of momentum to the surrounding pore-fluid molecules [66]. The soil zeta potential, defined as the electrical potential existing at the junction between the fixed and mobile parts of the electrical double, is influenced by the type and concentration of dissolved ions in the pore fluid in addition to the pore fluid chemistry. Clay soils, being negatively charged, usually possess negative zeta potential. At low pH below the point of zero charge (PZC), zeta potential may become positive because of excessive protonation and increase in ionic strength resulting from increased dissolution of metal ions in the pore fluid and their subsequent adsorption onto the soil particles and compression of the electrical double layer [67]. Reversal of the zeta potential charge could reverse the direction of the electroosmotic velocity as shown in (10). At high pH values, such as those encountered in this study, deprotonation and metal hydroxide precipitation could maintain a negative zeta potential; hence, electroosmotic flow will remain unidirectional as observed in all the tests. Electroosmotic flow has not been influenced by hydraulic gradient in this study as it occurs even under negative hydraulic. Equation (11) presents the model equation (*R* ^2^ = 0.946 and adjusted *R* ^2^ = 0.9057) relating the electroosmotic volume to the factors. Voltage gradient appears to be the most influential, followed by polarity reversal rate and initial contaminant concentration (Figure 8(a)). At high voltage gradient (1 V/cm), the decrease in the electroosmotic volume (Figure 8(b)) may be attributed to the development of bubbles within the electrode chambers, due to temperature rise, which then seeps into the soil to reduce the soil saturation with subsequent reduction in the electroosmotic volume [24]:

(11) Sqrt  (Electroosmotic  volume,mL) =49+2.57∗A  +11.68∗B+1.22∗C  +5.26∗B∗C−5.34∗A2  −20.95∗B2.

**Figure 9:**
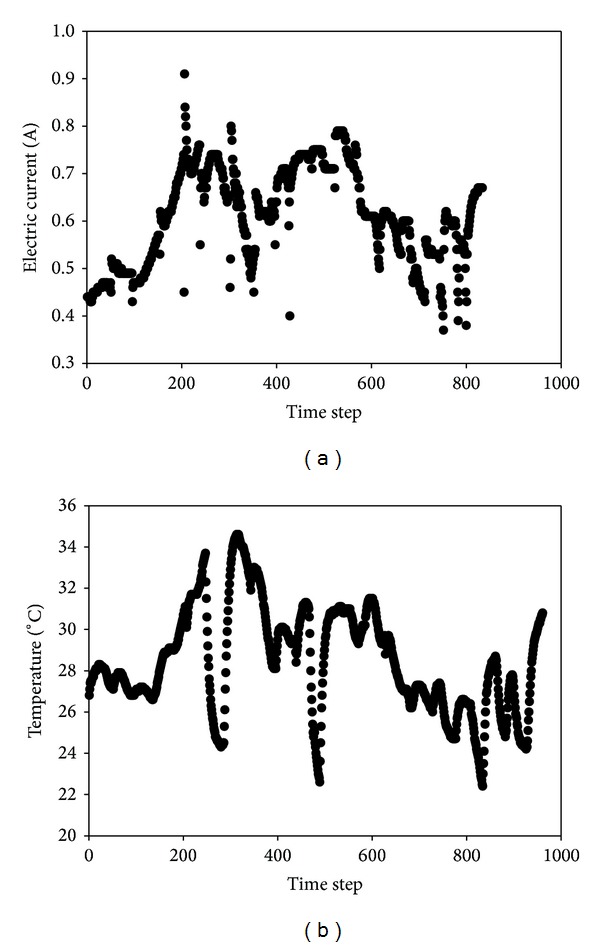
Comparing variations of electric current with soil temperature: (a) current; (b) temperature.

**Figure 10:**
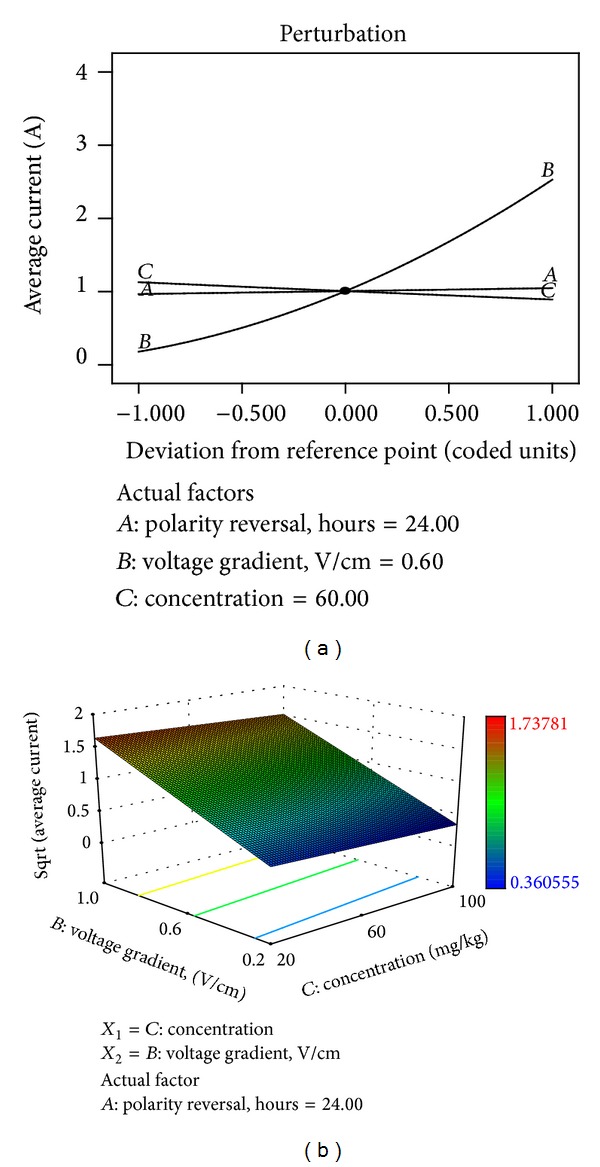
(a) Perturbation plot showing the relative significance of factors on average electric current. (b) 3D response surface and contour plots showing the influence of voltage gradient on average electric current.

*Current and Temperature*.  Table 5 presents the average electric current recorded for each test during the 3-week test duration in descending order of magnitude to show how it is influenced by the applied voltage gradient and how it correspondingly affects the soil pH. Clearly, the higher the voltage gradient, the more amount of current is passed through soil which results in rapid generation of H^+^ and OH^−^ ions and subsequent rise in soil pH (Table 5). The current is usually low at the beginning of the tests (Figure 9(a)), rises gradually as the tests continue, and then declines, sometimes to a stable value, while in some instances, keeps on fluctuating according to the time-dependent geochemical processes taking place such as ionic dissolution and precipitation and degradation of the processing fluids. Study conducted by Maturi and Reddy [68] corroborated the fluctuating current trend. Upon application of the driving force, the voltage gradient, the processing fluids, and pore fluid migrate while the dissolved ions electromigrate to opposite poles. These processes lead to increase in the ionic strength of the pore fluid thereby increasing the current flow to a maximum value. The observed decline of the current to a stable value may be attributed to the electromigration of cations and anions to the respective electrode with subsequent possible precipitation of the cations due to increase in the soil pH as the test progresses [66, 69]. Temporal geochemical processes such as mineral and chemical dissolution and neutralization reactions taking place in the electrode chambers also contribute to the variation of the electric current. A maximum value of 5.13 A was recorded for R6 whose average current was 3.02 A. This current is considered extremely high, considering the fact that it is about two orders of magnitude greater than the recorded current values for other bench-scale studies that employed the Lasagna process (<30 mA) in other soil apart from saline-sodic soil as shown in Table 1. Other studies using electrokinetic remediation only using voltage gradient of 1 V/cm or higher have reported higher values but usually less than 300 mA [58, 66, 70]. Using low voltage gradient of 0.2 V/cm has only resulted in reducing the current to about 130–210 mA (Table 5). This unique and important observation may be explained by the high salinity and sodicity of the investigated soil which provides large amount of dissolved salts and minerals (carbonates) in the pore fluid for sustained high electrical conduction. High current flow through the soil will significantly affect the soil temperature, electroosmotic flow rate, electrode material and processing fluids degradation, soil pH, geochemical processes, remedial efficiency and energy consumption. In a related study by Lukman et al. [24], they also recorded similar high current (2.8 A). To emphasize on the effect of the electric current on the soil temperature, current and temperature readings recorded using a time step of 30 min is presented in Figure 9 for R11 (voltage gradient = 0.6 V/cm). This test has 0.61 A and 28.45°C as the average current and temperature respectively. The maximum values were 0.91 A and 34.6°C respectively which were recorded under room temperature of 24°C. It is clear from Figure 9 that low current leads to low soil temperature and vice-versa. In a preliminary study conducted by Lukman et al. [24] using 1 V/cm, 36.34°C, and 47°C were the average and maximum soil temperatures, indicating that the soil becomes very hot when using 1 V/cm. While soil heating may be advantageous in increasing the volatility of organics, solubility of minerals (carbonates), and reduction in pore fluid viscosity which will increase electroosmotic flow, it may also be undesirable since it will reduce the soil moisture content due to pore fluid evaporation with subsequent reduction in current and electroosmotic flow. In addition, it will increase soil electrical conductivity and energy expenditure [20]. Previous studies have not reported significant rise of soil temperature during bench-scale tests [20]. A linear model was obtained (12) which relates the factors to the average electric current whose respective* R* ^2^ and adjusted* R* ^2^ are 0.9556 and 0.9435. The perturbation and response surface plots (Figure 10) also revealed the significant influence of the applied voltage gradient over initial contaminant concentration and polarity reversal rate:

(12) Sqrt  (Average  current) =1+0.020∗A+0.59∗B−0.059∗C.

### 3.3. Trivalent Chromium Migration, Model Validation, and Optimization

**Figure 11:**
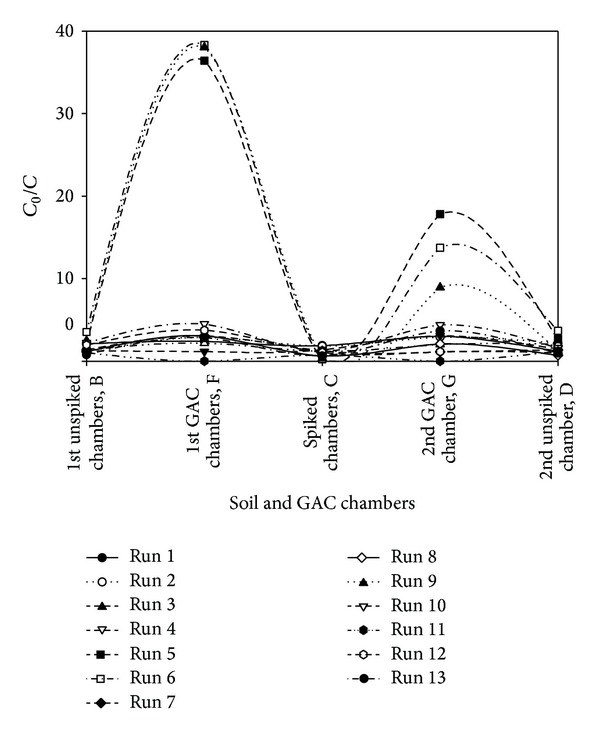
Trivalent Cr distribution and migration from the contaminated chamber to the GAC chambers after 13 tests.

**Figure 12:**
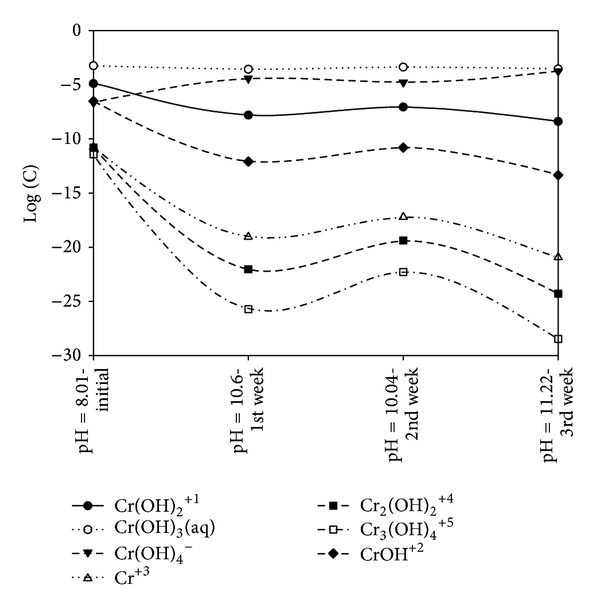
Speciation diagram for trivalent Cr species at different weekly pH values.

Figure 11 presented the distribution and migration of trivalent Cr from the contaminated chamber, C, to the GAC chambers F and G for all the thirteen (13) tests. This migration becomes more pronounced for tests R5, R6, and R9. In the case of R6 (no polarity reversal), significant trivalent Cr migration took place from the contaminated chamber, C, to the GAC chamber, F, near the anode. This observation may be attributed to the formation of high amount of negatively charged metal hydroxocomplexes at pH 12.9, which are then attracted to the anode via electromigration but become adsorbed onto the GAC in chamber F during the transport process. Visual MINTEQ 3.0 [51] was employed to model the trivalent Cr ion speciation for R5 from the weekly monitoring data using the dissolved concentration, pH, temperature and ionic strength. The speciation diagram presented in Figure 12 reveals the increasing dominance of the negatively charged complex Cr(OH)_4_^−^ and the decreasing concentration of aqueous Cr(OH)_3_ at pH 11.2. This explains the greater movement of the trivalent Cr species toward the anode in R6 at pH 12.9. Pourbaix [71] and Chinthamreddy and Reddy [29] have already asserted that Cr(OH)_4_^−^ ions will become the dominant species at pH values greater than 11.8, thus, trivalent Cr solubility increases. However, under normal soil pH, trivalent Cr has limited solubility and highly adsorbs to soil [29, 32]. In a related study by Reddy and Chinthamreddy [30] which involved an alkaline and high acid buffering soil called glacial till, they did not observe significant trivalent Cr migration and no removal. Although, the soil redox state may be dynamic because of the generation of oxygen and hydrogen gases at the electrodes in addition to the possible presence of iron (reducing agent), manganese (oxidizing agent) or microorganisms that can oxidize the trivalent Cr to the hexavalent form; oxidation of trivalent Cr does not take place appreciably in high buffering capacity soil such as saline-sodic soil [28]. For this reason, hexavalent Cr was not studied. Migration of the trivalent Cr from the contaminated chamber to the GAC chambers indicated remarkable remedial efficiency for some of the tests (R5, R6 and R9) while others indicated low or no removal at all (R1–R4, R7, R10, and R12). There is zero remedial efficiency when there was accumulation of the contaminant at the sampling location thereby having the residual concentration (*C*_o_) to be greater than the initial (*C*), in which case,* C*_o_/*C* > 1. Hence Figure 11 utilized* C*_o_/C to indicate the migration of trivalent Cr when* C*_o_/*C* < 1 or its accumulation at any given location or chamber when* C*_o_/*C* > 1.

Mass balance analyses of Cr were performed for Runs 8, 11, and 13. From Table 6, the mass balance for Runs 8, 11, and 13 is 121.75, 74.51, and 148.99%, respectively. These values were obtained using the ratio between the residual Cr in the contaminated chamber (C) plus any increase in Cr concentration in the GAC chamber and the initial Cr concentration. Among other reasons for the discrepancies in mass balance that is sometimes encountered during electrokinetic remediation as put forward by previous investigators [30, 66, 72] include adsorption onto the electrode and geotextile materials (which houses the GAC in the two chambers) and non-uniform distribution of contaminants within the small soil sample (about 2 g) taken for acid digestion and analysis. Taking different samples spatially from the contaminated chamber will help improve the mass balance.

**Figure 13:**
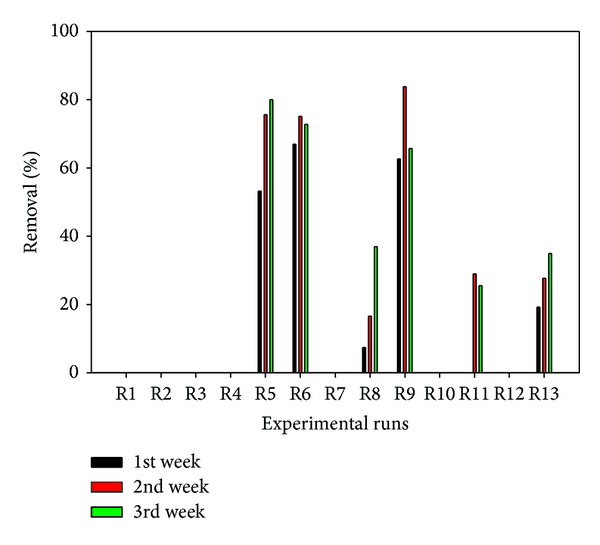
Weekly percentage removal of trivalent Cr for 13 tests.

The tests were sorted in decreasing order of remedial efficiency (Table 7) to reveal some salient points that will help in providing adequate connection between factors and responses. Highest remedial efficiencies (79.97–34.88%) were recorded for tests involving 60 mg/kg initial trivalent Cr concentration, whereas no removal was recorded for all tests involving 20 mg/kg. Only one test involving 100 mg/kg recorded some remedial efficiency (Table 7). Low remedial efficiency at 20 mg/kg may be attributed to the availability of adsorption sites for trivalent Cr ions coupled with the high selectivity for Cr for this particular soil type [45] at the given concentration. At higher concentrations (100 mg/kg) and pH, trivalent Cr may precipitate as Cr(OH)_3_, thus, rendering it immobile [66]. Even with low electric current, electroosmotic flow and voltage gradient (0.2 V/cm), 34.88% and 36.93% of the trivalent Cr was removed from the contaminated chamber in tests R13 and R8, respectively. Polarity reversal rate did not show any discernible pattern. Hence, there is need for simultaneous optimization of these three factors for optimal removal of the trivalent Cr. It is important to note that high voltage gradient (1 V/cm) or passage of high electric current does not necessarily translate into high remedial efficiency but will definitely increase the energy expenditure. At high voltage gradient, current is high, leading to high electroosmotic flow toward cathode. This opposite flow may interfere with the electromigration of the anionic trivalent Cr species that are migrating toward the anode, thus, reducing the overall remedial efficiency. Electromigration constitute the major transport mechanism for charged species whose rate is 10–300 times higher than the advective electroosmotic transport [73]. At low voltage gradient (0.2 V/cm), extremely low electroosmotic flow takes place and sustained electromigration prevails. The weekly percentage removal of trivalent Cr from the contaminated chamber is presented in Figure 13. The dynamic and temporal changes in the geochemical processes controlling the contaminant removal are attributable to the observed trends in the weekly percentage removal.

Equation (13) relates the investigated factors to the remedial efficiency with 0.9335 and 0.8966 as the* R* ^2^ and adjusted* R* ^2^ values, respectively:

(13) Sqrt  (Cr,remedial  efficiency) =8.78−0.71∗A+0.58∗B  +0.63∗C−1.50∗B2−7.39∗C2.

**Figure 14:**
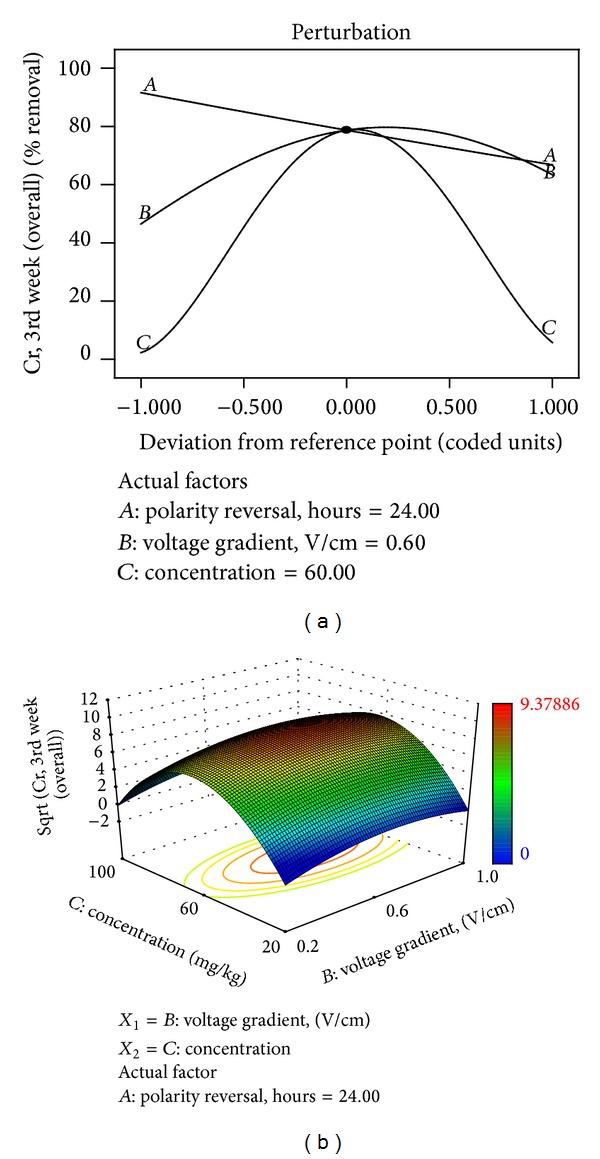
(a) Perturbation plot showing the relative significance of factors on trivalent Cr remedial efficiency. (b) 3D response surface and contour plots showing the influence of initial contaminant concentration on trivalent Cr remedial efficiency.

Perturbation plot (Figure 14(a)) also supports the observed influence of the initial Cr concentration on the remedial efficiency, followed by voltage gradient, then, polarity reversal rate. The investigated factor levels can be used to determine the optimal conditions required to achieve maximum remedial efficiency as depicted in the 3D response surface plot (Figure 14(b)).

*Model Validation*. To validate the practical applicability of the developed models affecting the remedial efficiency (13) and soil pH (9), additional experimental test was run at voltage gradient of 1 V/cm, initial contaminant concentration of 44.15 mg/kg, and without polarity reversal (Table 8). Results of the model validation showed that the experimental results lie within 90% confidence interval (CI) and prediction interval (PI) with associated prediction error of 2.35% and 32.64% for soil pH and remedial efficiency, respectively. Since the validation results fall within the prediction interval, then, the outcome of the confirmation test was a success [39]. Hence, the models can provide good approximations necessary to move in the proper direction.

**Figure 15:**
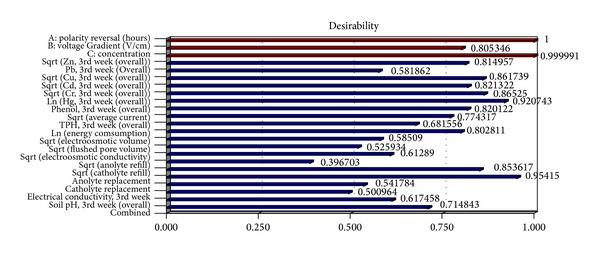
Combined and individual response desirability values for all responses and factors.

**Figure 16:**
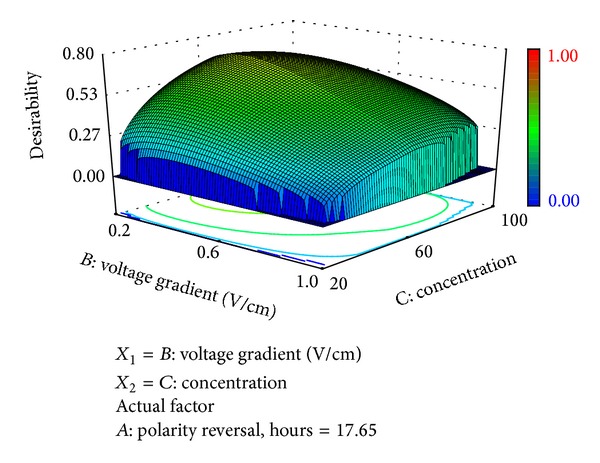
3D surface plot of the overall desirability variation relative to influential factors.

*Optimization of Trivalent Chromium Removal*. Numerical optimization was employed to find the optimal factor levels that will specifically target maximum remedial efficiency of trivalent Cr while optimizing all the other contaminant remedial efficiencies and responses (Figure 15). An overall desirability value of 0.715 was obtained and its variation based on the influential factors (initial concentration and voltage gradient) is depicted in Figure 16. Optimal conditions required to achieve effective trivalent Cr removal at 60 mg/kg are presented in Table 9. Overall desirability of 0.715 was attained at the following optimal conditions: voltage gradient = 0.36 V/cm; polarity reversal rate = 17.63 hr; soil pH = 10.0. Under these conditions, the expected trivalent Cr remedial efficiency is 64.75%.

### 3.4. Impacts of the Integrated Electrokinetic Remediation on Soil Physicochemical Properties

Preceding sections have elaborately discussed and modeled the impacts of the proposed remediation technique on the soil pH and electrical conductivity. Additionally, the passage of electric current and soil pH gradients will result in the following physicochemical interactions: (1) possible dissolution of the clay minerals beyond a pH range of 7–9; (2) dissolution of available soil salts such as carbonates; (3) production of cementitious products resulting from the precipitation of metal ions at pH values corresponding to their hydroxide solubility values; and (4) soil structural changes which affect its engineering characteristics [41–44]. Surface area, pore volume and size (Table 10), mineralogical compositions (Table 11), and elemental constituents (Table 12) were analyzed, before and after the test for R5. At the end of the test (pH = 11.2), the soil specific surface area has increased (9.07 to 11.21 m^2^/g) with corresponding increase in the pore volume and size. These results have confirmed that some dissolution of the soil minerals has taken place during the electrokinetic remediation process due to variations in the pore fluid chemistry. Soil pores are due to the presence of interlayer spaces that becomes prominent in 2 : 1 clay mineral types such as montmorillonite and smectite [40, 62, 74]. Table 11 presents the mineral transformation where dolomite completely disappeared; calcite and quartz were altered and degraded, respectively, after the test. The constituent soil elements were not spared as the amount of each one either increased or decreased after the test as shown in Table 12. These observations may be explained by microbially-driven biotransformation processes involving dissolution and precipitation, which take place under both aerobic anaerobic conditions. This leads to mineral dissolution and formation of new minerals from aqueous ions (biomineralization) as noticed in Table 11 [40]. Yong et al. [40] have asserted that the scientific basis for biomineralization is still not well understood.

## 4. Conclusions

The study reported herein investigated the migration of trivalent Cr ions from a multiple contaminated natural saline-sodic soil. The soil salinity and sodicity, which provided large amount of dissolved salts and minerals (carbonates) in the pore fluid for sustained high electrical conduction, were responsible for the extremely high electric current flow. This led to excessive soil heating, high energy and process fluid consumption, high electroosmotic volume, and in some cases higher percentage removal of trivalent Cr. Significant migration of Cr from the contaminated chamber to the granular activated carbon chamber was recorded which led to highest remedial efficiencies (79.97–34.88%) for tests involving 60 mg/kg initial trivalent Cr concentration, whereas no removal was recorded for all tests involving 20 mg/kg. Even under low electric current, electroosmotic flow, and voltage gradient (0.2 V/cm), up to 36.93% of the trivalent Cr was removed from the contaminated chamber. It has been shown that high voltage gradient (1 V/cm) or passage of high electric current does not necessarily translate into high remedial efficiency. Bipolar effects did not manifest due to the presence of carbonate minerals that impact high acid buffering capacity. For test without polarity reversal, trivalent Cr moved toward the anode due to the formation of high amount of anionic Cr(OH)_4_^−^ hydroxocomplex at high pH, which was further attracted to the anode via electromigration. Nonadsorption of this ion onto the negatively charged clay soil due to the possession of similar charge increased its availability and mobility. Speciation modeling using Visual MINTEQ 3.0 reveals the increasing dominance of the anionic Cr(OH)_4_^−^ and the decreasing concentration of aqueous Cr(OH)_3_ at pH 11.2. Effects of voltage gradient, initial contaminant concentration, and polarity reversal rate on the effective removal of Cr ions were experimentally studied using the Box-Behnken Design of experiment and mathematically modeled and numerically optimized using response surface methodology. Results of the model validation showed that the experimental results lie within 90% confidence interval and prediction interval with associated prediction error of 2.35% and 32.64% for soil pH and trivalent Cr remedial efficiency, respectively. Overall desirability of 0.715 was attained at the following optimal conditions: voltage gradient = 0.36 V/cm; polarity reversal rate = 17.63 hr; and soil pH = 10.0. Under these conditions, the expected trivalent Cr remedial efficiency is 64.75%. Passage of electric current and variations in the pore fluid chemistry led to soil mineral dissolution and alteration via biotransformation.

## Figures and Tables

**Table 1:** Applications of Lasagna process at bench-scales from inception to date.

Treatment zone material	Contaminant	Soil type	Cell dimensions (length × width × depth)	Polarity reversal/ downtime	Removal efficiency, %	Voltage gradient, (V/cm)/ current (mA)	Power consumption, kWhr/m^3^	Run time, days	Electroosmotic conductivity, cm^2^ V^−1^ s^−1^ (×10^−5^)	Treatment zone spacing, cm	Reference
AC∗ + sand, bacteria + AC + sawdust	*p*-nitrophenol	Kaolinite	10 cm ID, 21.6 cm long	Yes/ continuous	90–99	1–7/3 (constant)	10	20	2.5	6	[14, 20]

AC (Bamboo charcoal)	Cd	Sandy loam	24 cm × 10 cm × 8 cm	Yes/ continuous	79.6	1/7–27	—	12	—	10	[21]

AC (Bamboo charcoal)	Cd	Kaolin	24 cm × 10 cm × 8 cm	No/ continuous	93	1/3–23	—	8	—	10	[22]

AC (Bamboo charcoal)	2,4-dichlorophenol and Cd	Sandy loam	24 cm × 10 cm × 10 cm	Yes/ continuous	75.97 (Cd); 54.92 (2,4-dichlorophenol)	1/Variable	121.91–128.48	10.5	—	16	[23]

GAC∗∗	Cr, Cd, Cu, Pb, Hg, Zn, phenol, kerosene	Saline-sodic clay	24 cm × 10 cm × 12 cm	No/ continuous	75.9 (Cr); 34.4 (Cd); 41 (Cu); 55.8 (Pb); 92.49 (Hg); 26.8 (Zn); 100 (phenol); 49.8 (kerosene)	0.6–1/880	1777–4273	21	425	6	[24]

*Activated carbon; ∗∗Granular activated carbon.

**Table 2:** Applications of Lasagna process at pilot- and field-scales from inception to date.

Treatment zone material	Contaminant	Soil type	Site dimensions (length × width × depth)	Polarity reversal/ downtime	Removal efficiency, %	Voltage gradient, (V/cm)/Current (A)	Power consumption, kwh/m^3^	Run time, month	Electroosmotic conductivity, cm^2^ V^−1^ s^−1^(×10^−5^)	Treatment zone spacing, cm	Reference
AC + sand	*p*-nitrophenol	Kaolin/ Kaolinite/ clay loam	1.22 m × 0.61 m × 0.61 m	Yes/ continuous	98	1 (constant)/96.2 (based on current density)	51	3	0.56–1.7	35.56	[20]

GAC^1^	TCE^2^	Clay loam	4.6 m × 3 m × 4.6 m	Yes/ continuous	99	0.35–0.45/40 (constant)	—	4	1.2	60	[25]

Iron filings + kaolin	TCE	Clay loam	6.4 m × 9.2 m × 13.7 m	Yes/3-week	95–99	0.23–0.31 (constant)/ 110–200	—	12	1.2	60 & 150	[26]

Iron filings + kaolin	TCE	Clay loam	27.4 m × 22 m × 13.5 m	No/pulse mode	99	0.15–0.26 (constant)/ 500–700	—	24	—	150	[15, 27]

Iron filings + kaolin	TCE	Clay loam	33 m × 24 m × 7.5 m	—	60 (after 1 year)	0.16 (constant)/ 250–400	—	24	—	150	[15]

^1^Granular activated carbon; ^2^Trichloroethylene.

**Table 3:** Codification and ranges of factors.

Variable	Designation	Units	Coded variable levels		
			−1	0	+1
Polarity reversal	*A*	hr	0	24	48
Voltage gradient	*B*	V/cm	0.2	0.6	1
Concentration	*C*	mg/kg	20	60	100

**Table 4:** Design of experimental runs using the Box-Behnken design.

Run order	Polarity reversal, *A* (hr)	Voltage gradient, *B* (V/cm)	Concentration, *C* (mg/kg)	Remedial efficiency, %
1	0	0.6	20	0.00
2	48	0.6	20	0.00
3	24	1	20	0.00
4	24	1	100	0.00
5	24	0.6	60	79.97
6	0	1	60	72.73
7	24	0.2	20	0.00
8	0	0.2	60	36.93
9	48	1	60	65.66
10	48	0.6	100	0.00
11	0	0.6	100	25.50
12	24	0.2	100	0.00
13	48	0.2	60	34.88

**Table 5:** Comparing electrical current with voltage gradient and soil pH for all tests.

Run	Current, A	Voltage gradient, V/cm	pH
R6	3.02	1	12.9
R9	2.65	1	12.6
R3	2.25	1	12.6
R4	2.04	1	12.7
R2	1.32	0.6	10.9
R1	1.17	0.6	10.2
R10	1.12	0.6	11.9
R5	1.03	0.6	11.2
R11	0.61	0.6	12.0
R8	0.21	0.2	9.8
R12	0.15	0.2	8.3
R7	0.14	0.2	8.1
R13	0.13	0.2	10.4

**Table 6:** A sample mass balance analysis of trivalent Cr for Runs 8, 11, and 13.

Runs	Run 8	Run 11	Run 13
Initial concentration, mg/kg	37.20	77.95	37.20
Residual concentration, mg/kg	23.46	58.08	24.23

1st GAC chamber, F			
Initial concentration, mg/kg	6.90	6.90	6.90
Residual concentration, mg/kg	21.15	0.00	19.70

2nd GAC chamber, G			
Initial concentration, mg/kg	6.90	6.90	6.90
Residual concentration, mg/kg	14.48	0.00	25.30

Mass balance, %	121.75	74.51	148.99

**Table 7:** Comparing trivalent Cr remedial efficiency with factors and some responses.

Runs	Remedial	Current,	Residual,	Electroosmotic volume,	Polarity reversal	Voltage gradient,	Initial Cr
	efficiency, %	A	pH	mL	rate, hr	V/cm	concentration, mg/kg
R5	79.97	1.03	11.2	2344.50	24	0.6	60
R6	72.73	3.02	12.9	1201.50	0	1	60
R9	65.66	2.65	12.6	1399.50	48	1	60
R8	36.93	0.21	9.8	81.00	0	0.2	60
R13	34.88	0.13	10.4	63.00	48	0.2	60
R11	25.50	0.61	12.0	1387.84	0	0.6	100
R1	0.00	1.17	10.2	1728.00	0	0.6	20
R2	0.00	1.32	10.9	2542.50	48	0.6	20
R3	0.00	2.25	12.6	814.50	24	1	20
R4	0.00	2.04	12.7	2272.50	24	1	100
R7	0.00	0.14	8.1	396.00	24	0.2	20
R10	0.00	1.12	11.9	2236.50	48	0.6	100
R12	0.00	0.15	8.3	324.00	24	0.2	100

**Table 8:** Experimental validation of trivalent Cr remedial efficiency and soil pH using voltage gradient = 1 V/cm; average concentration = 44.15 mg/kg; and polarity reversal rate = 0 hr.

Response	Experimental result	Model prediction	Prediction error, %	90% CI∗	90% CI	90% PI∗∗	90% PI
				low	high	low	high
Cr, remedial efficiency	75.88	51.11	32.64	31.17	75.95	18.36	100.00
Residual soil, pH	12.3	12.6	2.35	11.7	13.5	10.8	14.0

*Confidence interval.

∗∗Prediction interval.

**Table 9:** Optimal factor levels required to maximize remedial efficiency of trivalent Cr.

Item	Value
Polarity reversal, hours	17.63
Voltage gradient, V/cm	0.36
Concentration, mg/kg	60.00
Expected remedial efficiency of trivalent Cr	64.75
Expected residual soil pH	10.00
Desirability	0.715

**Table 10:** Values of soil surface area and pore volume and size, before and after treatment.

Description	BET∗ surface area,	Pore volume,	Pore size,
	m^2^/g	cm^3^/g	Å
Before	9.07	0.014	62.55
After	11.21	0.045	163.24

*BET: Brunauer-Emmett-Teller.

**Table 11:** Soil mineralogical transformations before and after treatment.

Phase name	Before, %	After, %
Quartz, SiO_2_	87.4	55.3
Calcite, CaCO_3_	5.2	44.7
Dolomite, CaMg(CO_3_)_2_	7.4	—

**Table 12:** Values of constituent soil elements, before and after treatment.

Element	Before, %	After, %
Ca	37.64	42.06
Si	34.73	23.42
Fe	10.41	15.06
Al	7.6	9.55
K	3.42	4.61
Mg	2.48	2.49
Pd	2.85	1.46
Ti	0.86	1.35
